# The Multiple Roles of the Cytosolic Adapter Proteins ADAP, SKAP1 and SKAP2 for TCR/CD3 -Mediated Signaling Events

**DOI:** 10.3389/fimmu.2021.703534

**Published:** 2021-07-06

**Authors:** Nirdosh Dadwal, Charlie Mix, Annegret Reinhold, Amelie Witte, Christian Freund, Burkhart Schraven, Stefanie Kliche

**Affiliations:** ^1^ Institute of Chemistry and Biochemistry, Freie Universität Berlin, Berlin, Germany; ^2^ Institute of Molecular and Clinical Immunology, Health Campus Immunology, Infectiology and Inflammation (GCI^3^), Medical Faculty of the Otto-von-Guericke University, Magdeburg, Germany; ^3^ Coordination Center of Clinical Trials, University Medicine Greifswald, Greifswald, Germany

**Keywords:** T-cell activation, integrin signaling, ADAP/SKAP1/2, T cells, adaptive immunology

## Abstract

T cells are the key players of the adaptive immune response. They coordinate the activation of other immune cells and kill malignant and virus-infected cells. For full activation T cells require at least two signals. Signal 1 is induced after recognition of MHC/peptide complexes presented on antigen presenting cells (APCs) by the clonotypic TCR (T-cell receptor)/CD3 complex whereas Signal 2 is mediated *via* the co-stimulatory receptor CD28, which binds to CD80/CD86 molecules that are present on APCs. These signaling events control the activation, proliferation and differentiation of T cells. In addition, triggering of the TCR/CD3 complex induces the activation of the integrin LFA-1 (leukocyte function associated antigen 1) leading to increased ligand binding (affinity regulation) and LFA-1 clustering (avidity regulation). This process is termed “inside-out signaling”. Subsequently, ligand bound LFA-1 transmits a signal into the T cells (“outside-in signaling”) which enhances T-cell interaction with APCs (adhesion), T-cell activation and T-cell proliferation. After triggering of signal transducing receptors, adapter proteins organize the proper processing of membrane proximal and intracellular signals as well as the activation of downstream effector molecules. Adapter proteins are molecules that lack enzymatic or transcriptional activity and are composed of protein-protein and protein-lipid interacting domains/motifs. They organize and assemble macromolecular complexes (signalosomes) in space and time. Here, we review recent findings regarding three cytosolic adapter proteins, ADAP (Adhesion and Degranulation-promoting Adapter Protein), SKAP1 and SKAP2 (Src Kinase Associated Protein 1 and 2) with respect to their role in TCR/CD3-mediated activation, proliferation and integrin regulation.

## Introduction

After T-cell maturation in the thymus, naïve CD4+ and CD8+ T cells navigate into secondary lymphoid organs (e.g. lymph nodes and spleen). In the lymph node, T cells migrate into the T-cell zone where they scan and interact with APCs such as dendritic cells (DCs). The TCR (T-cell receptor)/CD3 complex of CD8+ T cells recognizes peptide-loaded MHC (Major Histocompatibility Complex) class I molecules on DCs and enables the differentiation of naïve CD8+ T cells into CTLs (cytotoxic T lymphocytes). CTLs main function is to kill virus-infected cells and tumor cells [for reviews please see (1, 2)]. Activation of naïve CD4+ T cells is initiated when their TCR/CD3 complex detects foreign antigenic peptides presented on MHC II molecules by DCs. Depending on the surrounding cytokine milieu, CD4+ T cells subsequently differentiate into Th (T helper) subtypes, for instance Th1, Th2 or Th17 cells. These Th subtypes have various properties that enable them to fight against pathogens, to activate B cells for antibody production or to recruit/activate innate immune cells [for reviews please see (3, 4)].

**Figure 1:**
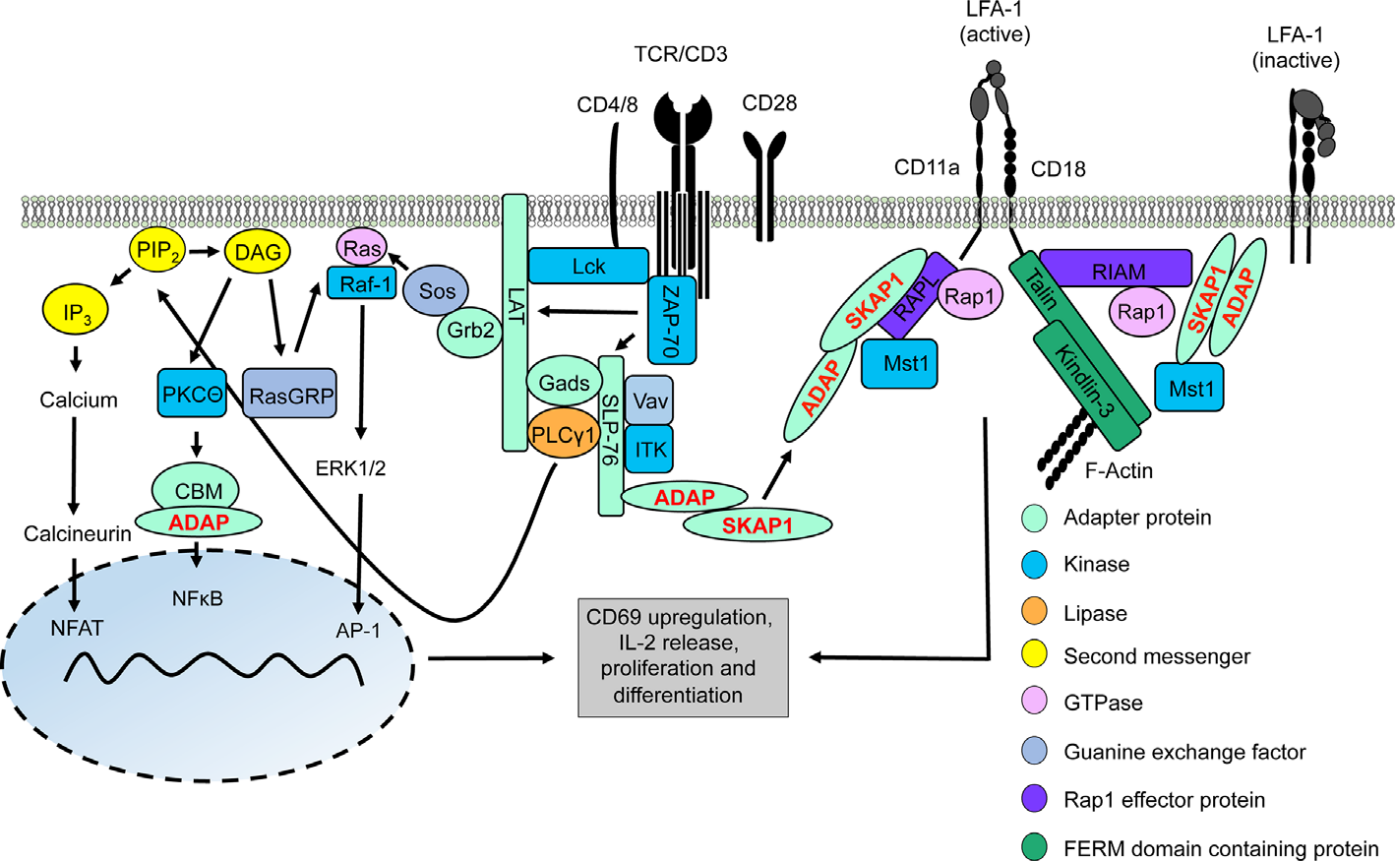
TCR/CD3 machinery leading to gene regulation and integrin activation. Upon TCR/CD3 complex stimulation, activated Lck phosphorylates ITAMs of the CD3 complex allowing recruitment and activation of ZAP-70. Active ZAP-70 phosphorylates the adapter proteins LAT and SLP-76, thus facilitating the formation of the LAT signalosome (includes LAT, SLP-76, Gads, Grb2, PLCγ1, ITK, ADAP, and SKAP1). ITK phosphorylates PLCγ1 which subsequently hydrolyses PIP_2_ into DAG and IP_3_. IP_3_ induces a signaling pathway for activation of the transcription factor NFAT into the nucleus whereas DAG activates two different transcription factors, NFκB (via the CBM/ADAP complex) and AP1 (Ras/ERK1/2 pathway). Translocation of these transcription factors into the nucleus facilitates T-cell activation and synthesis of IL-2 to support T-cell proliferation and differentiation. At the same time, two LFA-1-activating signaling complexe with the ADAP/SKAP1-modules as core elements are recruited to the cytoplasmic tails of LFA-1 promoting its activation for ICAM-binding. One LFA-1-activating signaling complexe binds to the cytoplasmatic domain of the LFA-1 α-chain (CD11a) chain consists of ADAP/SKAP1/RAPL/Rap1/Mst1 while the other complex is associated to the cytoplasmatic domain of the LFA-1 β-chain (CD18) contains ADAP/SKAP1/RIAM/Mst1/Rap1 connecting Talin and Kindlin-3 to LFA-1 and F-actin.

The simultaneous binding of the TCR/CD3 complex to peptide loaded MHC complexes (signal 1) and of CD28 to CD80/CD86 expressed by APCs (signal 2) is required for T-cell activation (termed co-stimulation). The TCR/CD3 complex consists of the TCRαβ heterodimer, the CD3ϵδ and CD3ϵγ heterodimers and the ζζ homodimer (here referred as TCR/CD3). The TCR/CD3-induced downstream signaling is dependent on phosphorylation of particular tyrosine motifs called ITAMs (immunoreceptor tyrosine-based activation motifs) located within the cytoplasmic domains of the CD3ϵ- and ζ-chains (5–7). Lck (lymphocyte-specific protein tyrosine kinase) and Fyn (feline yes-related protein), two members of the Src family of protein tyrosine kinases phosphorylate the ITAMs upon T cell activation. The precise mechanisms how Lck and Fyn propagate the TCR/CD3-mediated signal is still under intense debate (8–11). Nevertheless, following ITAM-phosphorylation, the tyrosine kinase ZAP-70 (ξ-chain associated protein of 70 kDa), a member of the Syk (spleen tyrosine kinase) family, is recruited to the phosphorylated ITAMs *via* its tandem SH2 domain and becomes activated by Lck. The adapter protein LAT (linker for activation of T cells) becomes subsequently phosphorylated by ZAP-70. Recently Lo et al., discovered that Lck promotes ZAP-70 activation for LAT phosphorylation through the formation of a molecular bridge between LAT and ZAP-70 (12) (**Figure 1**). In addition to LAT phosphorylation ZAP-70 phosphorylates SLP-76 (SH2 domain-containing leukocyte phosphoprotein of 76 kDa) and both adapter proteins form a signaling hub called the LAT signalosome. This signalosome drives gene expression, cytoskeleton re-organization and T-cell activation [for reviews please see (13–15)] (**Figure 1**).

The LAT signalosome connects the TCR/CD3 complex to the major downstream signaling pathways that regulate T-cell activation and T-cell proliferation. Crucial interaction partners of the LAT signalosome include the cytosolic adapter proteins ADAP (Adhesion and Degranulation-promoting Adapter Protein) and SKAP1 (Src Kinase Associated Protein 1), the tyrosine kinase ITK (interleukin T cell kinase), the guanine nucleotide exchange factor Vav and phospholipase Cγ1 (PLCγ1). Activation of PLCγ1 is of particular importance, since upon TCR/CD3 stimulation, PLCγ1 hydrolyzes the membrane lipid PIP_2_ (phosphoinositole-4,5 bisphosphate) to generate IP_3_ (inositol-1,4,5-triphosphate) and DAG (diacylglycerol) (16). These so called second messengers lead to the activation of three transcription factors – NFAT (nuclear activated T cells), NFκB (nuclear factor kappa light chain enhancer of activated B cells) and AP1 (activator protein 1), which control T-cell activation (like CD69 upregulation), IL-2 production/release and T-cell proliferation (13–15) (**Figure 1**).

The second messenger IP_3_ activates IP_3_ receptors in the membrane of the ER (endoplasmic reticulum) to induce calcium efflux from the ER. The reduced calcium levels within the ER promote conformational changes of STIM 1 and STIM 2 (stromal interaction molecules 1 and 2). Subsequently, STIM1/2 homodimers translocate to ER-plasma membrane junctions and interact with ORAI1 (Calcium Release-Activated Calcium Modulator 1). ORAI1 together with ORAI2 and ORAI3 form CRAC (calcium release activated channels) at the plasma membrane. The formation of the CRACs enables the entry of extracellular calcium into the cytosol (17). Here, calcium activates the serine phosphatase calcinuerin, which in turn dephosphorylates NFAT allowing the translocation of this transcription factor to the nucleus (18).

In contrast to IP3, the second messenger DAG activates the serine kinase PKCθ (protein kinase C theta) which phosphorylates CARMA1 (CARD-containing MAGUK protein 1). Subsequently, and in concert with the cytosolic adapter protein ADAP (see below), phosphorylated CARMA1 recruits Bcl10 (B-cell CLL-lymphoma 10) and MALT1 (mucosa-associated lymphoid tissue lymphoma translocation gene 1) to assemble the CBM (CARMA1-Bcl10-MALT1) complex (19). The CBM complex (20, 21) activates IκB kinase (IKK). Subsequently, activated IKK phosphorylates IκBα (inhibitor of kappa B) which induces release of IκBα from NFκB. While phosphorylated IκBα becomes polyubiquitinated and degraded in the proteasome, activated NFκB translocates to the nucleus where it initiates transcription of NFκB target genes (22) (**Figure 1**).

Finally, activation of the transcription factor AP1 depends on the Ras-Raf-ERK pathway. Ras (rat sarcoma) is a small GTPase that is activated by the guanine nucleotide exchange factors RasGRP1 (Ras guanyl-releasing protein 1) and Sos (son of sevenless) (23). Activated Ras binds and activates the serine kinase Raf-1 (rat fibrosarcoma) at the plasma membrane. Activated Raf-1 leads to phosphorylation and activation of the dual specificity kinases ERK1 and 2 (extracellular signal-regulated kinase) which then translocate to the nucleus where they phosphorylate AP1. Activated AP1 controls the transcription of different genes and drives together with NFκB CD69 expression, commonly used as a marker for T-cell activation (13, 24).

Besides inducing the above described signaling events, triggering of the TCR/CD3 activates integrins. Integrins are heterodimeric transmembrane receptors consisting of one α and one β chain. Each αβ subunit contains an extracellular domain, a transmembrane domain, and an unstructured cytoplasmic tail (**Figure 1**). T cells express members of the β1-chain containing VLA family (very late antigen; α4β1 (VLA-4, CD49d/CD29), α5β1 and α6β1) and the β2-integrin LFA-1 (leukocyte function-associated antigen-1; αLβ2 or CD11a/CD18). Ligands for VLA-4 are VCAM-1 (vascular cell adhesion molecule 1) and the matrix protein fibronectin (25). Binding of VLA-4 to its ligands is required for T-cell adhesion to the extracellular matrix and migration of T cells to sites of inflammation (26, 27). The ligands of LFA-1 are ICAM-1-5 (intercellular adhesion molecule 1-5) (25, 28). Ligand binding between LFA-1 and ICAM-1-5 is important for T cell adhesion on endothelial cells of the high endothelial venules, migration of T cells into peripheral lymph nodes and T-cell interaction with APCs (29).

On resting T cells, LFA-1 exists in a closed conformation (bend conformation) which possesses a very low affinity for its ligand, ICAM-1. In this state, the extracellular, ligand-binding headpiece bends down to the plasma membrane and the cytoplasmic domains of the α- and β-chains of LFA-1 are in close proximity to each other (**Figure 1**). Ligation of TCR/CD3 induces a conformational change in LFA-1 resulting in changes in affinity and avidity for its ligands (see below). The high affinity state of LFA-1 is characterized by an exposed/open headpiece and separated cytoplasmic domains of the α- and β-chains (**Figure 1**). Active LFA-1 also forms clusters at the cell surface. This process has been termed avidity regulation and represents another way to increase ligand binding and to strengthen the interaction of activated T cells with APCs. The signaling pathways leading to affinity/avidity modulation of LFA-1 have collectively been termed “inside-out signaling” (25, 29–31). Ligand bound LFA-1 transmits a signal into the cell that participates in T-cell activation (e.g. CD69 and CD25 upregulation and IL-2 production), T-cell proliferation and T-cell differentiation (e.g. Th1 cells) (31–34). This process is called “outside-in signaling” (25, 29–31). Hence, ICAM-1-bound LFA-1 acts as an accessory signal to enhance T-cell activation (35). However, recent data suggested that affinity regulation of LFA-1 promotes T-cell activation whereas clustering of this integrin terminates adhesion [(36), see below].

One negative regulator and several positive regulators and have been reported to modulate TCR/CD3-induced LFA-1 activation. Filamin A has been described to serve as a negative regulator for LFA-1 activation (37–39). Prior to T-cell activation Filamin A interacts with the cytoplasmic tail of the LFA-1 β-chain thereby keeping LFA-1 in an inactive conformation. However, *via* an as yet unknown mechanism, stimulation of the TCR/CD3 activates the serine/threonine kinase Ndr2 (nuclear Dbf2-related 2), which phosphorylates Filamin A at serine (S) 2152 and induces its dissociation from the LFA-1 β-chain. The release of Filamin A from LFA-1 now allows binding of positive regulators of LFA1-activation, e.g. Talin and Kindlin-3 (see below) (37) (**Figure 1**).

In contrast to filamin A, loss of the FERM (4.1 Protein, Ezrin, Radixin, Moesin)-domain containing proteins Talin and Kindlin-3 interfere with TCR/CD3-mediated LFA-1 activation (40–45). Talin and Kindlin-3 bind to distinct sites within the cytoplasmic tail of the LFA-1 β-chain and stabilize its open (active) conformation (46, 47).

Next to Talin and Kindlin-3, ADAP and SKAP1 constitutively interact with each other and form the ADAP/SKAP1-module. The ADAP/SKAP1-module is the core component for the assembly of two independent pools of LFA-1-activating signaling complexes (48). Co-immunoprecipitation studies had shown, that either the Rap1 effector proteins RIAM (Rap1-GTP interacting adapter molecule) or RAPL (Regulator of cell Adhesion and Polarization enriched in Lymphoid tissue) constitutively associate with the ADAP/SKAP1-module *via* binding to SKAP1 (49–51). Both RAPL and RIAM bind the activated GTPase Rap1 (Ras-proximity-1) (52, 53). Loss of these Rap1 effector proteins or of Rap1 in T cells attenuates TCR/CD3-mediated LFA-1 activation (45, 53–56). Besides binding to activated Rap1, RAPL interacts with the serin/threonine kinase Mst1/2 (mammalian Ste20-like kinase 1/2) in T cells. Similar to RAPL, Mst1/2 deficient T cells display reduced TCR/CD3-mediated adhesion to ICAM-1 (55–57).

Two LFA-1-activating signaling complexes are recruited to the plasma *via* interaction to the cytoplasmic domains of LFA-1. One LFA-1-activating signaling complex contains the ADAP/SKAP1/RAPL-module including Mst1 and binds *via* RAPL to the α-chain (CD11a) of LFA-1 (**Figure 1**). In contrast, the second LFA-1-activating signaling complex consists of the ADAP/SKAP1/RIAM-module and is linked to Kindlin-3, Talin as well as Mst1 and binds to the LFA-1 β-chain (CD18) [(48), **Figure 1**]. In addition both Kindlin-3 and Talin possess binding motifs for the interaction with filamentous (F)-actin [(58, 59) **Figure 1**].

It is important to note, that, in addition to plasma membrane recruitment *via* binding to the cytoplasmic domains of LFA-1, the two ADAP/SKAP1-modules are recruited to the phosphorylated LAT signalosome *via* the Gads/SLP-76-complex (60–62). Indeed, loss of SLP-76 in T cells attenuates plasma membrane targeting of ADAP and SKAP1 and impairs LFA-1 activation (62). In this review, we focus on recent findings on the ADAP and SKAP adapter proteins for TCR/CD3-mediated signaling events that regulate activation, proliferation and integrin-mediated inside-out and outside-in signaling in T cells.

## ADAP

**Figure 2:**
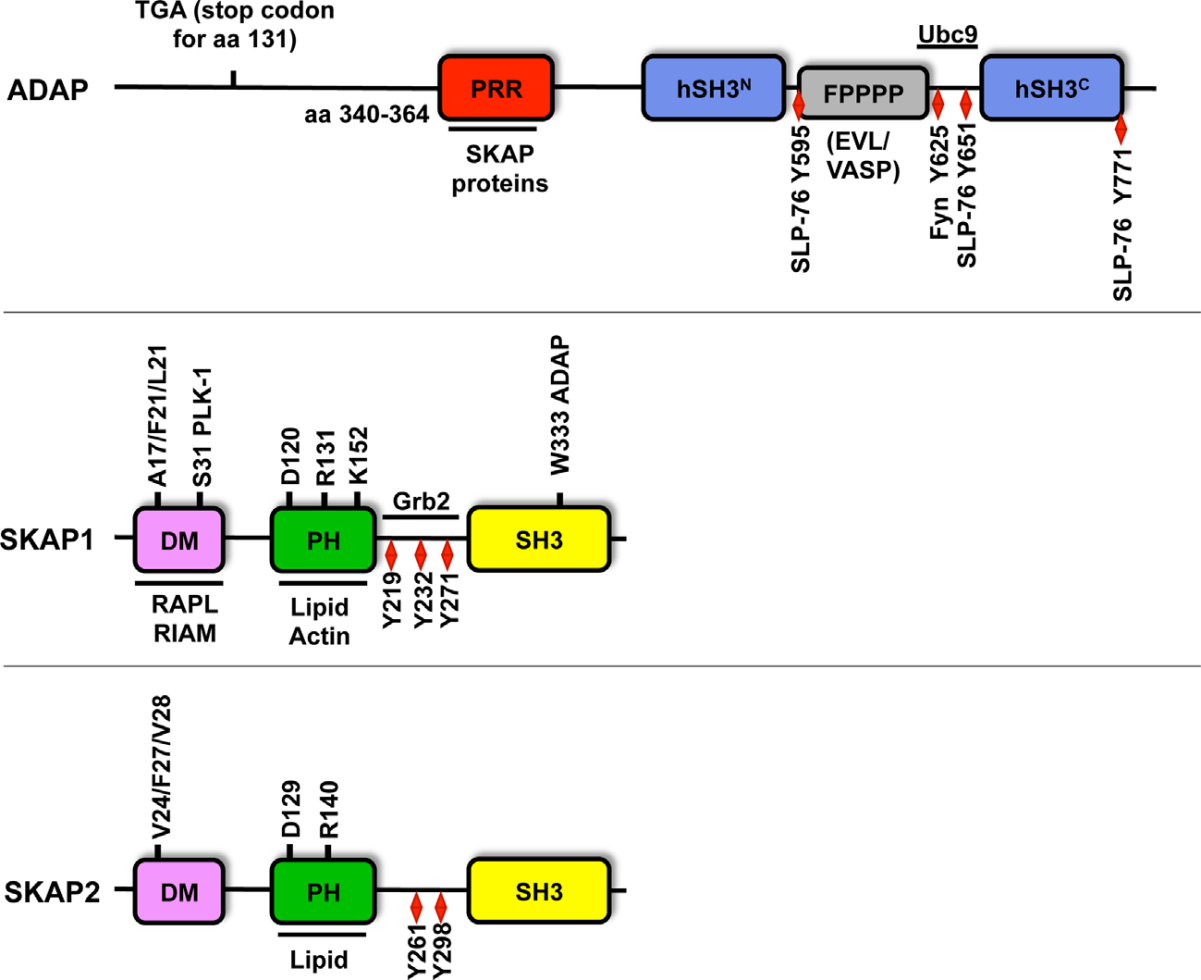
Structure of ADAP, SKAP1 and SKAP2. Amino acid (aa) numbering is given for human ADAP (short isoform NP_955367.1), SKAP1 (NP_003717.3) and SKAP2 (NP_003921). ADAP possesses an unstructured N-terminal region (containing the predicted stop codon at position 131 of CARST patients with a *Fyb* gene defect), a proline rich region (PRR), two helical SH3 domains (hSH3), an Evl/VASP binding site (FPPPP), a NLS motif (binding site for Ubc9) and multiple tyrosine motifs (Y). Amino acid 340-364 of the PRR of ADAP binds to W333 within the C-terminus Src homology domain (SH3) of SKAP1. Both SKAP proteins possess a dimerization domain (DM), a Pleckstrin homology domain (PH) and a SH3 domain. SKAP1 contains three tyrosine motifs (red diamond), but SKAP2 possess only two of these motifs. Both SKAP proteins contain three amino acids at position V24/F27/V28 for SKAP2 or A17/F20/L21 for SKAP1 in the DM domain, which are important for dimerization. The amino acids R131/140 and K152 within the PH domain contribute to lipid and actin binding and are required for plasma membrane targeting. The amino acids D120/129 are critical residues for the DM-PH auto-inhibition model (see **Figure 3**). The S31 in the dimerization domain is involved in cell cycle regulation *via* PLK-1 binding. Other binding partners of individual amino acids and domains are depicted.

ADAP [alias SLAP-130 (SLP-76-associated protein of 130kDa (60)], or FYB [Fyn-binding protein (63)] is expressed in thymocytes, peripheral T cells and other hematopoietic cells [for review please see (64)]. As depicted in **Figure 2**, ADAP possess a non-structured N-terminal region, a proline-rich region (PRR), two helical SH3 (Src homology 3) domains, a FPPPP-motif that mediates binding to members of the Ena (Enabled)/VASP (Vasodilator-stimulated Phosphoprotein) family and several tyrosine-based signaling motifs [for review please see (65)]. Knockout and knockdown studies had shown early on that ADAP is a positive regulator of T-cell development, TCR/CD3-induced T-cell activation (CD69/CD25 upregulation), IL-2 and IFN-γ production, proliferation and LFA-1 affinity/avidity regulation [for reviews please see (27, 64–67)].

Initial studies only investigated the pan T-cell population of conventional ADAP-deficient mice (68, 69) and did not distinguish between CD4+ and CD8+ T-cells. In contrast, a recent study by Parzmair et al. focused on T-cell subset-specific roles of ADAP revealing no differences in the expression levels of ADAP protein between the two T-cell subsets in mice (70). However, following TCR/CD3 stimulation, early activation and proliferation of CD4+ T cells appear to be more dependent on ADAP compared to CD8+ T cells. In addition, TCR/CD3-induced adhesion to ICAM-1 seems to be controlled by ADAP in the CD4+ but not in the CD8+ T-cell subset (70).

To analyze antigen-specific proliferation, conventional ADAP knockout mice were crossed to OT-I and OT-II TCR transgenic mice whose T cells recognize specific peptides of the model antigen ovalbumin. Upon breeding of ADAP knockout mice with OT-II mice, nearly no CD4+ T cells matured in the thymus whereas ADAP-deficient OT-I TCR transgenic CD8+ T cells showed normal T-cell maturation. Moreover, peripheral ADAP-deficient OT-I TCR transgenic CD8+ T cells showed no defects upon *in vitro* activation and proliferation in response to their cognate antigen peptide. The authors concluded that ADAP is critically involved in TCR/CD3 mediated signaling pathways for CD4+ T-cell development, whereas CD8+ T-cell maturation and functions are less dependent on ADAP (70). It is important to note at this point that, in contrast to TCR/CD3-mediated signals, ADAP appears to be required in both T-cell subpopulations to propagate chemokine receptor mediated signals (70). However, the molecular mechanisms, how ADAP regulates chemokine receptor mediated pathways is still elusive.

### ADAP and NFκB Activation

It is well established that ADAP is crucial for the assembly of the CBM complex (20, 21). However, it has been shown that murine ADAP-deficient CD4+ T cells do not display a complete defect in TCR/CD3/CD28-induced NFκB activation (20, 21). This might be due to the fact that TCR/CD3 and CD28 use two independent pathways to activate NFκB in T cells (71, 72). Thus, TCR/CD3-induced NFκB activation *via* the CBM complex formation is ADAP-dependent, whereas CD28-mediated signaling activates NFκB in an ADAP-independent fashion *via* Grb2 (growth factor receptor-bound protein 2) and Vav1.

### ADAP and SLP-76

Signaling *via* the TCR/CD3 complex depends on the formation of the LAT signalosome whose core elements are LAT and SLP-76. In imaging studies SLP-76 was visualized in structures termed SLP-76 microclusters, which contain many other signaling molecules/complexes. Defects in SLP-76 microcluster persistence and movement correlate with attenuated proximal TCR/CD3-mediated signaling events, including calcium release and CD69 upregulation (73, 74). Previous studies had shown that the C-terminal SH2 domain of SLP-76 is crucial for microcluster formation, suggesting that this domain facilitates incorporation of other signaling molecules/complexes into the microclusters (73). Three tyrosine phosphorylation sites (Y595, Y651 and Y771) in ADAP were identified that are involved in the interaction with the SH2 domain of SLP-76 (61, 75, 76). Mutations of either two (Y595 and Y651) or all three tyrosine residues abrogated TCR/CD3-induced adhesion, interactions with APCs and integrin clustering (avidity regulation) [(76–78), see also **Figure 1**]. Coussens et al. provided experimental evidence that phosphorylation of any combination of two of the three tyrosine phosphorylation sites within ADAP facilitates multipoint binding and thereby allows its oligomerization with SLP-76. Hence, the formation and persistence of SLP-76 microclusters appears to depend on an interaction between SLP-76 and the ADAP/SKAP1 signaling modules (51, 76).

The study by Coussens et al. was substantially extended by detailed quantitative analysis of ADAP containing SLP-76 microcluster movement and persistence (79). ADAP exists in two isoforms: -the 120 kDa isoform is preferentially expressed in the thymus, while the longer 130 kDa isoform predominates in mature T cells (80). Lewis et al. provided evidence that both isoforms of ADAP move to TCR/CD3-induced SLP-76 microclusters (79). In addition, the multivalent interactions between tyrosine Y595 and Y651 of ADAP and the SH2 domain of SLP-76 were confirmed in this study (78). The interaction between ADAP and SLP-76 stabilized the phosphorylation of ADAP at Y595 as analyzed by Western blotting using a phospho-Y595-specific antibody (79). Surprisingly, deletion of the N-terminal region of ADAP reduced the phosphorylation at Y595 and disrupted SLP-76 microcluster formation. Importantly, this ADAP deletion mutant not only lacks the non-structured N-terminal region but also the PRR motif. It is well established that this motif mediates the constitutive interaction between ADAP and SKAP1 (please see **Figure 2**) and stabilizes SKAP1 protein expression (70, 81, 82). Thus, it seems that the interaction between SKAP1 and ADAP is mandatory for ADAP phosphorylation at Y595 and SLP-76 microcluster formation. Indeed, Ophir et al. showed that knockdown of SKAP1 expression attenuated SLP-76 microcluster formation independently of ADAP in T cells (51).

Microscopic analyses revealed a second pool of ADAP that is not phosphorylated at Y595 and colocalizes with F-actin-rich lamellipodia (79). Lewis et al. postulated that the association of non-Y595-phosphorylated ADAP within lamellipodia pre-positions ADAP to potential TCR/CD3/MHC/peptide contact sites, thus enabling the rapid assembly of SLP-76 microcluster nucleation and their transport to the center of the immunological synapse after TCR/CD3 stimulation. In light of these data, the authors suggested a revised model of TCR/CD3 signaling where ADAP is not a simple downstream effector of SLP-76 but where both molecules function in parallel pathways that i) intersect at the level of SLP-76 microcluster formation and ii) promote TCR/CD3-induced adhesion *via* the Y595 phosphorylated ADAP/SKAP1-modul but is less important for the activation of NFκB (79).

### ADAP and Ubc9: A New Player for TCR/CD3-Mediated LFA-1 Activation

ADAP was the first adapter protein identified that is involved in TCR/CD3-induced inside-out and outside-in signaling events of LFA-1 (27, 65–67). Recently, Ubc9, the small ubiquitin-related modifier (SUMO) E2 conjugase was identified as an inducible binding partner of ADAP (83). Sumoylation is a multistep enzymatic process, which results in covalent attachment of SUMO to a lysine based consensus sequence in proteins. Amongst others, the biological function of sumoylation includes transcriptional regulation, nuclear transport and DNA repair (84). The multistep sumoylation process involves E1, E2, and E3 enzymes. Ubc9 is the sole E2 conjugating enzyme and depletion of Ubc9 leads to a blockade of the SUMO pathway in various cellular systems. Ubc9 knockdown studies in Jurkat T cells and in primary mouse T cells showed a reduced LFA-1 mediated adhesion to ICAM-1 upon TCR/CD3 stimulation (83). Co-immunoprecipitation studies further revealed an indirect interaction between Ubc9 and ADAP after TCR/CD3 stimulation (83). Using ADAP deletion mutants, the C-terminal NLS (nuclear localization site) was identified as binding site for Ubc9. This was the first description of a biological function of the NLS motif within ADAP in T cells (80). Mechanistically, downregulation of Ubc9 impaired TCR/CD3-mediated Rac1 activation and plasma membrane targeting of Rap1 and its effector protein RAPL Importantly, membrane targeting of RIAM was not attenuated. Interestingly, knockdown of Ubc9 had no impact on global tyrosine phosphorylation and IL-2 promotor activity in response to TCR/CD3stimulation. The authors concluded that Ubc9 is required for TCR/CD3-induced activation of Rac1, membrane targeting of Rap1 and RAPL and subsequent T-cell adhesion to ICAM-1. Further investigations are needed to clarify if and how an ADAP mutant lacking the C-terminal NLS for Ubc9 regulates plasma membrane recruitment of activated Rap1 bound to RAPL independently of RIAM to attenuate TCR/CD3-mediated adhesion to ICAM-1.

### ADAP and the Actin Cytoskeleton

Microscopic analyses of ADAP showing its colocalization with actin-rich lamellipodia structures [(79), see above] suggested that ADAP is also involved in remodeling of the actin cytoskeleton. Two independent signaling complexes have been described that link ADAP to processes facilitating actin-remodeling: the Nck-WASP complex or the Ena/VASP complex (78, 85–88). Pauker et al. proposed that the TCR/CD3-mediated interaction of Nck (Non-catalytic region of tyrosine kinase) with ADAP facilitates the association between SLP-76 and WASp (Wiskott-Aldrich syndrome protein) (88). Numerous reports show that T cells from WASp patients exhibit defective actin responses upon TCR/CD3 triggering [for review please see (89, 90)]. Similar to WASp deficient T cells, T cells lacking either ADAP or Nck revealed a partial impairment of F-actin dynamics, whereas knockdown of both ADAP and Nck showed severe defects in F-actin polymerization and lamellipodia formation (88).

The FPPPP motif within ADAP represents the binding region for proteins of the Ena/VASP family (**Figure 2**) (85). These proteins are regulators of the actin cytoskeleton. They control F-actin polymerization (91, 92) and reduce the length of F-actin filaments within lamellipodia structures (93). VASP and/or Evl (Ena-VASP-like) can associate with the ADAP/SKAP1-module by either directly binding to ADAP (85) and/or indirectly *via* RIAM (49, 53). Recently Estin and colleagues showed that T cells from Evl/VASP double knockout mice showed attenuated chemokine-mediated F-actin polymerization (94). Hence, it is likely that Ena/VASP proteins contribute to ADAP-mediated defects in F-actin polymerization and/or lamellipodia formation in response to TCR/CD3 stimulation leading to an attenuated LFA-1 activation and interaction of T cells with APCs.

### ARAP an ADAP-Homologue?

In 2016, Jung and colleagues identified a novel adapter protein termed ARAP (activation-dependent raft-recruited ADAP-like phosphoprotein) (95). The ARAP cDNA encodes for a protein of 728 amino acids with a predicted molecular weight of 83 kDa. ARAP shares sequence homology with ADAP: both proteins possess the N-terminal proline-rich region, conserved tyrosine-based signaling motifs and the N-terminal hSH3 domain. The binding site for Ena/VASP proteins (FPPPP), the N-terminal hSH3 domain and the putative nuclear localization sites (NLS) are unique for ADAP, whereas only ARAP contains an internal lysine-rich sequence. The mRNA of ARAP was found in various lymphoid tissues and the endogenous protein is expressed in Jurkat T cells. It was shown, that ARAP is required for TCR/CD3-mediated proximal signaling such as PLCγ1 activation and Ca^2+^ release, T-cell adhesion to ICAM-1 but not for TCR/CD3-mediated actin polymerization. In addition, interaction of ARAP with SLP-76, SKAP1, Lck and Fyn was demonstrated (95). Since confirmatory reports about ARAP are still missing, the biological significance of ARAP in T cells remains elusive.

## SKAP Proteins

The SKAP (Src Kinase-Associated Phosphoprotein) proteins includes SKAP1 (66) [also termed SKAP55 (Src Kinase-Associated Phosphoprotein of 55 kDa (96)] and its homolog SKAP2 (97) [also referred as SKAP-HOM (SKAP55-homolog (98)] or SKAP-55R [SKAP-55-related (99)]. Both proteins share the same domain composition such as a DM (Dimerization) domain, a PH (Pleckstrin Homology) domain and a C-terminal SH3 (Src Homology 3) domain. They share 44% identity on the protein level, mainly in their PH and SH3 domains (98, 99) (**Figure 2**). Human SKAP1 contains three tyrosine-based signaling motifs at amino acid position 219, 232 and 271 (51, 96) in the inter-domain, whereas human SKAP2 possess only two motifs at amino acid 261 and 298 (98) (**Figure 2**).

### SKAP1

SKAP1 is expressed in T lymphocytes (96, 100). The tryptophan 333 (W333) within the SH3 domain of SKAP1 interacts with the PRR motif of ADAP [see above, (81, 101, 102)]. Marie-Cardine and colleagues have shown that 70% of ADAP interacts with SKAP1 and depletion experiments further revealed that there is no free (=ADAP-unbound) SKAP1 protein present in T cells (101). Several studies subsequently demonstrated that loss of ADAP destabilizes SKAP1 leading to its degradation at the protein level while the mRNA levels remain unaffected (70, 81, 82). However, it is still unknown how ADAP protects SKAP1 from degradation. Huang et al. demonstrated that the half-life of SKAP1 drops from 90 minutes to 15 minutes in the absence of ADAP (81). They further suggested that ADAP either stabilizes a protease/caspase-resistant conformation of SKAP1 or that ADAP targets SKAP1 to subcellular compartments, which are less accessible to the proteases/caspase machinery (81). In summary, ADAP and SKAP1 form a functional unit and are here referred to as the ADAP/SKAP1-module. Knockout and knockdown studies of SKAP1 in T cells revealed that this adapter protein is involved in different TCR/CD3-mediated signaling pathways that regulate proliferation, IL-2 and IFN-γ production and LFA-1 avidity/affinity (27, 66, 67).

#### SKAP1 and SLP-76 Microclusters

Microscopic studies revealed that the ADAP/SKAP1-module is recruited into SLP-76 microclusters (51). Similar to ADAP-deficient Jurkat T cells, loss of SKAP1 attenuated SLP-76 microcluster persistence and movement (51). In this context SLP-76 microcluster stabilization was not affected upon mutation of the three tyrosine-based signaling motifs to phenylalanine within the inter-domain or deletion of the PH domain of SKAP1, but rather was dependent on the SH3 domain (the binding site for ADAP) and the DM domain (the binding site for RAPL or RIAM see **Figure 2**) within SKAP1. Deletion of the SH3- or the DM-domains within SKAP1 not only impaired SLP-76 cluster formation but also attenuated T-cell adhesion to fibronectin/ICAM-1 and interaction of T cells with APCs (49–51).

#### SKAP1 and Inside-Out/Outside-Signaling of LFA-1

The DM domain enables SKAP1 or SKAP2 homodimer formation (103, 104). Upon mutation of the three amino acids valine 24, phenylalanine 27 and valine 28 within the dimerization interface (V24/F27/V28), mutant SKAP2 failed to dimerize with wild type SKAP2. These three amino acids within the DM domain of SKAP2 correspond to alanine 17, phenylalanine 20, lysine 21 (A17/F20/L21) in the DM domain of SKAP1 (**Figure 2**). Similar to SKAP2, mutation of A17, F20 and L21 attenuated dimer formation of SKAP1. Surprisingly however, attenuated SKAP1 dimer formation did not interfere but rather enhanced RAPL binding. A possible explanation might be that dimerization limits SKAP1 binding to RAPL (or probably to RIAM). However, functional consequences of these mutations for TCR/CD3-mediated LFA-1 activation could not be analyzed due to low expression levels of the mutated protein in T cells (104).

As depicted in **Figure 2**, SKAP1 exhibits a central PH domain. PH domains are known to mediate protein/protein or protein/lipid [phosphatidylinositol (PI)] interactions that facilitate membrane targeting of signaling protein, including the SKAP proteins (105). We showed that the isolated PH domain of SKAP1 binds PIP_3_ [a lipid product generated by the PI3 (phosphatidylinositol-3) kinase (PI3K)] *in vitro* (106). Three amino acids, lysine 116 (K116), arginine 131 [R131, corresponding to R140 in SKAP2 (103)] and K152 were identified to be required for PIP_3_ binding *in vitro*. Localization studies in Jurkat and primary human T cells further revealed a constitutively associated plasma membrane targeting of the isolated PH domain of SKAP1. Surprisingly however, the constitutive membrane targeting of the PH domain was not affected following inhibition of PI3K by wortmannin or Ly294002 indicating that PIP_3_ localized at the plasma membrane is not required for membrane targeting. Surprisingly mutation of K152 within the PH domain to glutamic acid (K152E) completely interfered with plasma membrane targeting (106). Besides lipid-binding to PH domains, positively charged amino acids within these domains interact with actin as described for the PH domain of Btk (bruton’s tyrosine kinase) (107). Indeed, immunoprecipitation studies showed that the isolated PH domain of SKAP1 interacts with actin, whereas the K152E mutant failed to do so. This suggests that the isolated PH domain is targeted to the plasma membrane by a K152-mediated interaction with actin. However, co-sedimentation and co-precipitation assays using purified G (globular)- and F-actin revealed that the recombinant PH domain does not directly interact with actin (106). Therefore, identification of protein(s) that link the isolated PH domain of SKAP1 to actin requires further investigation. Possible candidates are the three members of the ezrin/radixin/moesin (ERM) family. In resting T cells, these proteins are located at the plasma membrane and are additionally linked to F-actin (108).

The functional relevance of the PH domain of SKAP1 for TCR/CD3-induced LFA-1 activation is controversially discussed in the literature (51, 82, 106, 109, 110). Burbach et al. and Raab et al. showed that deletion of the PH domain or mutation of R131 within the PH domain of SKAP55 impairs adhesion and conjugate formation of T cells with APCs (109, 110). In contrast, two other studies showed that neither deletion of the PH domain within full-length SKAP55 nor overexpression of the isolated PH domain of SKAP55 alters TCR/CD3-mediated adhesion (51, 82). However, mutation of K152E within full-length SKAP1 attenuated TCR/CD3-induced LFA-1 activation, adhesion and interaction of T cells with APCs. Of note, the defect in LFA-1 activation mediated by the K152E mutant was not due to impaired plasma membrane targeting of SKAP1 *via* the inducible interaction of ADAP with SLP-76 but rather on an attenuated assembly of RAPL to the α-chain and Talin/RIAM binding to the β-chain of LFA-1 (106). Why a single point mutation like K152E within the PH domain of full-length SKAP1 produces such a functional defect for TCR/CD3-mediated LFA-1 activation, whereas a deletion of the whole PH domain of SKAP55 does not alter integrin functions remains to be an unanswered question (106). No significant differences in the composition of the LFA-1-associated signaling molecules between full-length SKAP1 and deletion of the PH domain in SKAP1 expressing Jurkat T cells were observed in response to TCR/CD3 triggering (106). However, currently there is no model that would explain this contradicting and previously reported results that have been obtained by using deletion mutants of the PH domain versus a single point mutation (51, 82, 106).

**Figure 3:**
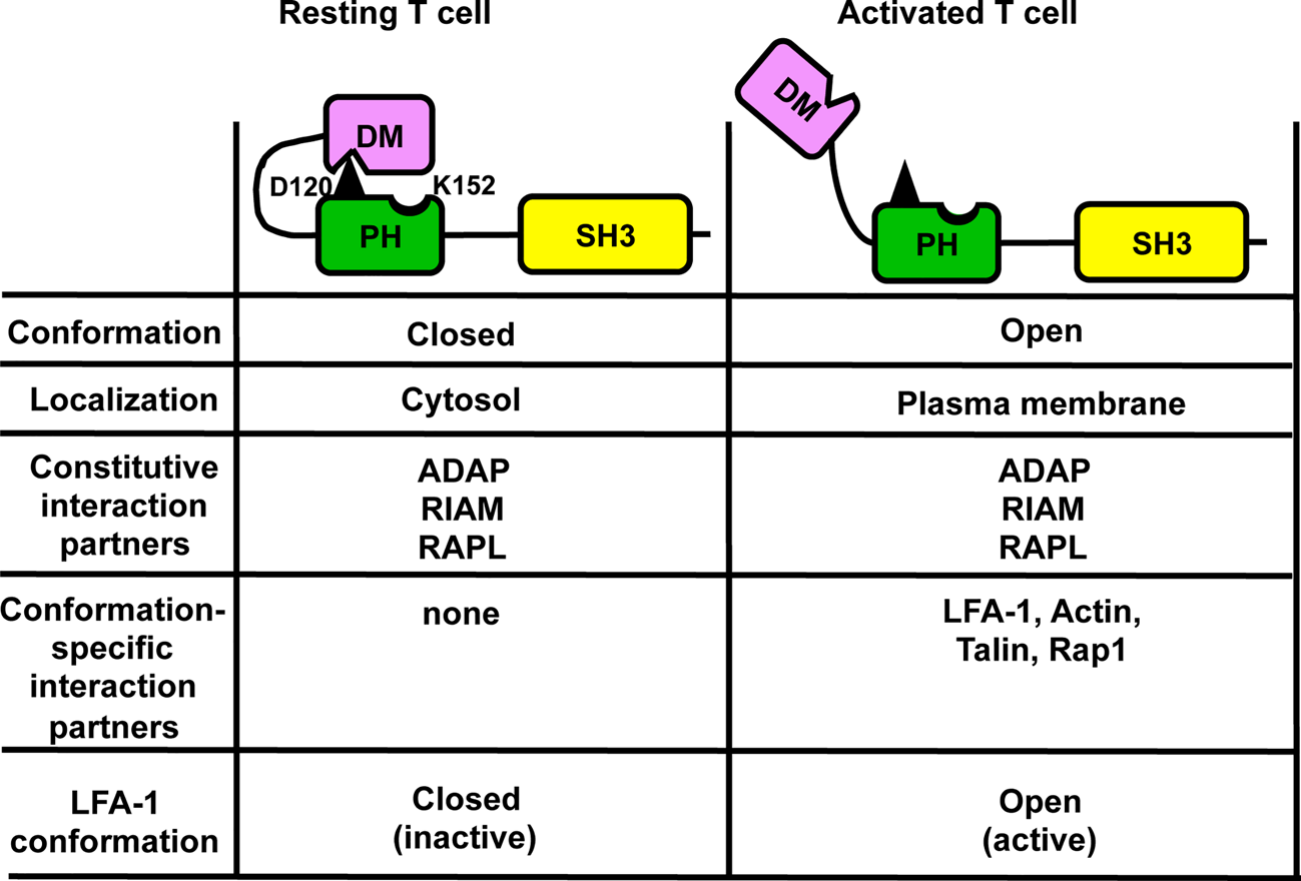
Auto-inhibition model of SKAP1 that controls targeting of SKAP1 to the plasma membrane. In resting T cells, SKAP1 is in a closed (auto-inhibited) conformation where aspartic acid 120 (D120; depicted in black) within PH domain mediates an interaction with the DM domain (DM-PH). In this conformation, SKAP1 is localized in the cytoplasm and is constitutively associated with ADAP, RAPL and RIAM. In TCR/CD3 -stimulated T cells, the DM-PH interaction is released by an unknown mechanism to stabilize the open (active) conformation, which enables recruitment of the constitutive interaction partners (ADAP, RAPL and RIAM) of SKAP1 to the plasma membrane. The now accessible lysine 152 (K152) within in the PH domain of SKAP1 inducible interacts with actin and promotes Rap1/Talin binding to RIAM and to LFA-1. Under these conditions LFA-1 is in an open conformation to facilitate adhesion.

In contrast to the constitutive plasma membrane localization of the isolated PH domain, full-length wild type SKAP1 primarily localizes in the cytosol in resting T cells (106) and only translocates to the plasma membrane upon T cell activation. Based on these observations it was proposed, that SKAP1 exists in two functional and conformational states: a closed, DM-PH auto-inhibited conformation that is located in the cytosol and an open conformation that localizes at the plasma membrane. That the DM and PH domains induce an auto-inhibited conformation was first shown for SKAP2 (103). Here, crystal structure analyses demonstrated that the isolated PH domain of SKAP2 (binds PIP_3_) has a different conformation compared to the PH domain within a DM-PH fragment. Within the DM-PH fragment, the β1-β2 loop of the PH domain adopts an open loop conformation and rearranges itself to a short helix that packs into a hydrophobic groove within the DM domain. The interaction of the PH domain of SKAP2 with the DM domain is predicted to represent an inhibited conformation that hinders binding to PIP_3_ (103). The aspartic acid at position 129 (D129) in SKAP2 (103) was identified to be responsible to mediate the closed DM-PH conformation. Indeed, mutating D129 to lysine (D129K) in SKAP2 induced a constitutive localization of the full-length protein to actin-rich membrane ruffles in macrophages (103). D129 of SKAP2 corresponds to D120 in SKAP1 (both localized within the PH domain; **Figure 2**) and, similar to the mutation D129K in SKAP2, mutation of D120K in SKAP1 resulted in constitutive membrane localization of full-length SKAP1 and induced a constitutive active state of LFA-1 in resting T cells (106). Importantly, these functional effects were abolished when an additional K152E mutation was introduced. Of note, in contrast to the D120K single mutant, the D120K/K152E double mutant was no longer able to interact with Talin, LFA-1 and actin. Consequently, it was proposed that an intra-molecular switch mechanism dynamically modifies the interaction of the N-terminal DM domain with the PH domain and enables the released PH domain of SKAP1 to localize to the plasma membrane and to initiate LFA-1 signaling events (**Figure 3**). These findings could bring SKAP1 in touch with RIAM and Talin, which are regulated in an auto-inhibitory manner for the TCR/CD3 -induced LFA-1 machinery (111, 112) and other adhesion-regulating molecules in various cell types (113).

Outside-in signaling of LFA-1 acts as a co-stimulatory signal for T-cell activation and differentiation (35). In the literature it was shown that the cytosolic adapter proteins SLP-76 and ADAP are also involved in LFA-1-mediated signaling events, indicating that the TCR/CD3 and LFA-1 use the two adapter proteins to initiate different signaling pathways (114–116). In this regard, Raab et al. recently showed that LFA-1 mediates both adhesion upon affinity induction and de-adhesion upon avidity regulation (clustering of LFA-1) (36). Cross-linking of LFA-1 (clustering) induced the phosphorylation of LAT on Y171 by members of the focal adhesion kinase family FAK-1 (Focal Adhesion Kinase-1) and PYK-1 (proline-rich tyrosine kinase-1). Phosphorylated Y171 acts as the binding site for a complex consisting of Grb2 and SKAP1 (and probably ADAP) (36). The SH3 domain of Grb2 was shown to interact with the inter-domain of SKAP1 (**Figure 2**), although the binding motif or the involved amino acids were not identified. The LAT/Grb2/SKAP1 complex appears to be distinct from the LAT/Gads (Grb2-related adapter downstream of Shc)/SLP-76 complex, which is required for TCR/CD3-induced calcium release, CD69 upregulation and LFA-1 activation (117). It was proposed that LFA-1 affinity regulation mediated contact/adhesion with APCs is followed by clustering of LFA-1 that subsequently terminates adhesion. The termination of adhesion by LFA-1 is mediated *via* activation of FAK-1, which phosphorylates LAT at Y171 and thus re-structures the LAT-signalosome (LAT/Grb2/SKAP1 complex) in T cells. Hence, the model proposes that LFA-1 is an “auto-regulatory on-off” receptor that can mediate adhesion and de-adhesion dependent on affinity *versus* avidity regulation of LFA-1 (36). However, until now, dynamic data are missing which would show a time dependent exchange of the LAT-associated Gads/SLP-76/ADAP-complex (mediated by TCR-activated ZAP-70) with a SLP-76-independent Grb2/SKAP1/ADAP-complex (induced by LFA-1 mediated activation of FAK-1).

#### SKAP1 and Cell Cycle Progression

SKAP1 is not only involved in TCR/CD3-induced LFA-1 activation, but also regulates T cell proliferation (118). In the study of Liu et al. it was stated, but not shown that SKAP1 and ADAP are localized in the nucleus of T cells (99). However, this observation prompted Raab and colleagues to test an array of kinases that are involved in proliferation for their ability to phosphorylate SKAP1 *in vitro*. The authors identified SKAP1 as substrate for the serin/threonine kinase PLK-1 (polo-like kinase-1) (119). PLK-1 is not expressed in resting but in proliferating T cells (120) where it regulates multiple stages of mitosis and cell cycle progression (121). PLK-1 phosphorylates serine 31 (S31) within the N-terminal dimerization domain of SKAP1. The interaction of SKAP1 with PLK-1 is needed for PLK-1 kinase activity to promote optimal cell cycling and growth of T cells (119). It seems that the SKAP1/PLK-1 complex is not needed for early T-cell activation events when LFA-1 activation takes place but rather participates in late signaling events when proliferation and DNA replication occurs in the nucleus. Of note, a similar multi-functional role at different subcellular locations has been described for SLP-76, which regulates TCR/CD3 proximal signaling pathways required for calcium mobilization or binding to RanGAP1 (Ran GTPase activating protein 1) to promote the transport of transcription factors into the nucleus of T cells (122).

### SKAP2

In contrast to SKAP1, SKAP2 is ubiquitously expressed, including T and B lymphocytes (98, 100). Similar to SKAP1, the SH3 domain of SKAP2 binds to ADAP and this interaction is essential for stable SKAP2 protein expression in T cells (82, 99). The role of SKAP2 has been studied using SKAP2-deficient mice (100). The expression levels of SKAP1 and ADAP are comparable in SKAP2-deficient and wild type T cells, indicating that SKAP2 does not interfere with SKAP1 stability. SKAP2-deficient B cells, which do not express SKAP1, showed reduced B cell receptor (BCR)-mediated proliferation and defective LFA-1 mediated adhesion or cluster formation (100). Strikingly, mature B cells do not express ADAP (100, 123) indicating that in wild type B cells another interaction partner stabilizes SKAP2 expression. One possible candidate could be RIAM that possesses two PRR regions and is also mandatory for BCR-mediated LFA activation (54). In contrast to B cells, SKAP2 knockout mice display no T-cell defects (100). The unaffected function of SKAP2 knockout T cells might be due to the ability of SKAP1, which is expressed in T cells but not in B cells, to compensate for the loss of SKAP2. Studies investigating the ability of SKAP2 to compensate for SKAP1 in T cells are controversial (51, 124). Thus, one study showed that knockdown of SKAP1 leads to attenuated LFA-1 clustering and impaired interaction of T cells with APCs that could not be reconstituted by expression of SKAP2 (124). In contrast, a second study by Ophir and colleagues revealed that when expressed at comparable levels, SKAP2 is able to rescue SLP-76 microcluster dynamics and T-cell adhesion to fibronectin in SKAP1 deficient cells (51). However, similar (but not as pronounced) as for ADAP-deficient mice, SKAP1-deficient T cells show attenuated proliferation, impaired IL-2/IFN-γ production and LFA-1 adhesion upon TCR/CD3 complex-stimulation (118). Hence, it would be interesting to find out, whether SKAP1/SKAP2 double knockout mice display comparable T-cell defects as reported for ADAP-deficient animals.

## Conclusion and Outlook

The three adapter proteins ADAP, SKAP1 and SKAP2 play crucial roles in the organization of different signalosomes at different subcellular locations and time points to regulate TCR/CD3-mediated signaling events for LFA-1 activation/deactivation and proliferation.

The core elements of the LAT signalosome are LAT and SLP-76. Genetic defects for LAT and more recently for SLP-76 in humans have been reported to cause severe immunodeficiencies and defective T-cell signaling (125, 126). A recent study by Levin et al. identified a homozygous mutation in the human *FYB* gene (that encodes ADAP). Importantly, and similar to ADAP-deficient mice, none of these patients developed primary immune defects or showed signs of unusual infections but all of them displayed small-platelet thrombocytopenia and an increased bleeding tendency (69, 127, 128). A c.393G>A mutation was detected that leads to the introduction of a stop codon (TGA) instead of tryptophan (TGG) at position 131 of the ADAP amino acid sequence [(128), **Figure 2**]. The introduction of this stop codon would interfere with all described functions of ADAP (SKAP1/2 expression, inducible interaction with SLP-76, LFA-1 activation and proliferation) in resting or stimulated T cells. Investigation of these patients’ lymphocytes would be helpful to address whether similar ADAP-dependent T-cell defects reported in mice exits in human T cells of these patients.

Several studies have used conventional ADAP-knockout mice to investigate the contribution of this adapter protein for infection (*Listeria monocytogenes* and H5N1 influenza virus) and diseases models [anti-tumor response, allogenic grafting and experimental autoimmune encephalomyelitis (EAE)]. T cells are essential for adaptive immune responses against pathogens and tumors and are involved in the immunopathogenesis of autoimmune diseases. However, besides its critical role in T cells, ADAP is expressed in a variety of immune cells of the innate immune system and in these cell types loss of ADAP interferes with various functions [for review see (64)]. Engelmann et al. had previously demonstrated that conventional ADAP knockout mice show strongly attenuated EAE (129). This was shown for active EAE as well as for passive EAE after adoptive transfer of activated TCR transgenic T cells specific for the MOG35-55 peptide (myelin oligodendrocyte glycoprotein-MOG) (129). In this disease model, myelin-specific CD4+ T cells are activated and expand in the peripheral lymphoid tissue; they cross the blood-brain barrier and enter the CNS. The inflammatory response leads to the recruitment of other immune cells including monocytes, macrophages, dendritic cells, B cells, and NK cells (130, 131). The invading monocytes, macrophages and dendritic cells express high amounts of MHC-II molecules and are involved in antigen presentation and reactivation of T cells within the CNS. In addition, resident microglia, monocytes and macrophages secrete pro- as well as anti-inflammatory cytokines depending on their environment and, furthermore, produce reactive oxygen species and nitric oxid (132). The inflammatory process leads to demyelination and axonal damage. All above-mentioned hematopoietic cells - T cells, NK cells, myeloid cells and platelets - express ADAP [for review see (64)]. Hence, analysis of conventional ADAP knockout mice cannot answer the question, which cell population contributes to the lower EAE severity in ADAP-deficient mice. To dissect the role of ADAP in different immune cell types during EAE, cell type specific ADAP-knockout mice were generated. ADAP was deleted in T cells, myeloid cells, NK cells and platelets using Cre recombinase under control of lineage specific promoters (133, 134). Afterwards, active EAE was induced in these animals by immunization with the MOG35-55 peptide. The clinical course of EAE was significantly milder in mice with loss of ADAP in T cells, myeloid cells and NK cells compared to ADAP-sufficient control littermates (133). Surprisingly, specific deletion of ADAP in platelets resulted in a more exacerbated disease (134). These findings indicate that in conventional ADAP knockout mice T cell-dependent and T cell-independent mechanisms are involved in the resistance to EAE.

## Author Contributions

ND was involved in writing of the introduction, section of SKAP proteins, generation of **Figures 1** and **2** and formatting of the manuscript. AR and CM wrote the ADAP part of this review. AW helped to generate **Figure 3**. SK, CF, and BS were involved in critical reading and/or writing of this manuscript. All authors contributed to the article and approved the submitted version.

## Funding

Funded by the Deutsche Forschungsgemeinschaft (DFG, German Research Foundation) RE-2907/2-2 (AR) and Project-ID 97850925 – SFB 854 (B12 (SK and CF) and B19 (BS)). BS is further supported by grants of the state of Saxony-Anhalt (SI2 and SI3).

## Conflict of Interest

The authors declare that the research was conducted in the absence of any commercial or financial relationships that could be construed as a potential conflict of interest.
